# A Comprehensive Study on Antibiotic Resistance among Coagulase-Negative Staphylococci (CoNS) Strains Isolated from Ready-to-Eat Food Served in Bars and Restaurants

**DOI:** 10.3390/foods12030514

**Published:** 2023-01-23

**Authors:** Wioleta Chajęcka-Wierzchowska, Joanna Gajewska, Anna Zadernowska, Cinzia Lucia Randazzo, Cinzia Caggia

**Affiliations:** 1 Department of Industrial and Food Microbiology, University of Warmia and Mazury in Olsztyn, Plac Cieszyński 1, 10-726 Olsztyn, Poland; 2 Department of Agriculture, Food and Environment (Di3A), Via Santa Sofia 100, University of Catania, 95123 Catania, Italy

**Keywords:** coagulase-negative staphylococci (CoNS), ready-to-eat food, antimicrobial resistance, methicillin resistant coagulase-negative staphylococci (MR-CoNS), multidrug resistant coagulase-negative staphylococci (MDR-CoNS)

## Abstract

The present study aimed to characterize and assess the diversity of CoNS strains as potential vectors for the spread of resistance to antimicrobial agents from RTE foods served in bars and restaurants. Eighty-five CoNS strains, obtained from 198 RTE food samples, were investigated. Sixty-seven CoNS isolates (78.8%) were resistant to at least one antibiotic tested, and 37 (43.5%) were multidrug resistant (MDR-CoNS). Moreover, CoNS strains contained genes conferring resistance to antibiotics critically important in medicine, i.e., β—lactams [*mecA* (29.4%); *blaZ* (84.7%)], aminoglycosides [*aac(6′)-Ie-aph(2″)-Ia* (45.9%); *aph(2″)-Ic* (3.5%)], macrolides, lincosamides and streptogramin B-MLS_B_ [*msrA/B* (68.2%); *ermB* (40%) and *mphC* (4.7%)], tetracyclines [*tetK* (31.8%); *tetM* (16.5%) and/or *tetL* (2.35%)]. We also found the *fus*B/C/D genes responsible for the acquired low-level fusidic acid resistance (17.6%) and streptogramin resistance determinant *vgaA* in 30.6% of isolates. In three linezolid resistant strains (2 *S. epidermidis* and 1 *S. warneri*), mutation was detected, as demonstrated by L101V and V188I changes in the L3 protein amino acid sequences. The high frequency in RTE food of MDR-CoNS including methicillin-resistant (MR-CoNS) strains constitutes a direct risk to public health as they increase the gene pool from which pathogenic bacteria can pick up resistance traits.

## 1. Introduction

Antimicrobial resistance (AMR), recently referred to as the “silent pandemic” [1], is a growing public health issue. Among antibiotic resistant bacteria, one of the biggest therapeutic challenges is widely recognized as methicillin-resistant staphylococci [2]. For many years, coagulase-positive *Staphylococcus aureus* has been considered the main pathogenic species amongst staphylococci; however, recent reports indicated the increasing role of coagulase-negative staphylococci (CoNS) in causing antibiotic-resistance infections. Currently, the most clinically relevant species are *Staphylococcus epidermidis* and *Staphylococcus heamolyticus* and, more recently, also *Staphylococcus saprophyticus* and *Staphylococcus lugdunensis* [3]. From a therapeutic perspective, CoNS pose a challenge due to the increasing occurrence of methicillin-resistant strains and the increasing isolation of strains with a lower susceptibility to glycopeptides [4]. Even though the detection and spread of multidrug-resistant (MDR) CoNS in the hospital setting is well documented, very few studies are related to the resistance of CoNS isolated from human related sources. According to the One Health approach, monitoring for the occurrence of antimicrobial resistant strains in different environments is widely recommended, mostly considering that antimicrobials are not used exclusively in medicine. As matter of fact, despite the legal prohibitions, antimicrobial agents continue to be used in prophylaxis or even metaphylaxis during livestock production [5]. Antibiotics, such as tetracycline and streptomycin, are used in the prophylaxis and in control of bacteria causing fruit infections [6]. Furthermore, in aquaculture, where antimicrobial doses applied may be higher than those prescribed for livestock, antimicrobial agent residues can be retained both in fish and in the aquatic environment. Such residues can be rapidly spread through feces in water bodies, where they exert selection pressure in the surrounding bacteria, sediment, and associated microbiota [7].

Considering the use of antimicrobials in the economic sectors related to food production, the number of reports on the occurrence of antibiotic-resistant CoNS in food has increased in recent years [8,9], strongly suggesting that food production chain may provide a route for the transmission of antimicrobial resistance genes. Besides the interest on food of animal origin, recently an increasing number of reports concerning ready-to-eat (RTE) foods confirmed their role as source of resistant strains. Previous studies, focused on staphylococci in RTE foods, mainly of animal origin, purchased at retail [10,11] have been performed. The present study focuses on RTE food served in bars and restaurants, where staphylococci may come from raw materials and from secondary contaminations, for which humans may play a considerable role. Staphylococci colonize the skin and mucous membranes of humans and are regarded as commensals or opportunistic pathogens [9]. Failure to observe proper hygienic conditions during food manipulation can result in their transfer to the food served to consumers. Therefore, the aim of this study was to characterize and assess the diversity of CoNS strains as potential vectors for the spread of resistance to antimicrobial agents from RTE foods served in bars and restaurants.

## 2. Materials and Methods

### 2.1. Isolation and Identification of CoNS by MALDI-TOF

Strains were isolated from 198 food samples including dishes (e.g., burgers, cheeses, juices, sushi, salads, sandwiches, meat and fish tatars) served in randomly selected (*n* = 11) bars and restaurants. All the isolation and identification steps were described previously [12]. Briefly, after preliminary phenotypic characterization, isolates negative for coagulase were identified by VITEK^®^ MS mass spectrometry microbial identification system with Matrix Assisted Laser Desorption Ionization Time-of-Flight (MALDI-TOF) (bioMérieux, Marcy l’Etoile, France), as previously described [13]. A mean spectrum for each isolate was constructed with at least 50 *m*/*z* spectra profiles and analyzed by the SARAMIS databases (version 4.13 RUO) (bioMérieux, Marcy l’Etoile, France). As relevant results of identification strains using MALDI-TOF for the species level considered a confidence level of ≥90%.

Additional typing using the *tuf* gene sequencing was performed for methicillin-resistant CoNS (MR-CoNS) to check the phylogenetic relationships. For *tuf* sequencing, genomic DNA was used for amplifying and sequencing an 827-bp fragment for presumptive *Staphylococcus warneri* isolates and an 830-bp fragment for other isolates, using tuf-F and tuf-R primers [14].

### 2.2. Phenotypic Antibiotic Resistance

An inoculum of CoNS isolates, adjusted to an optical density of 0.5 in McFarland scale, were swabbed onto the Mueller–Hinton agar (Merck, Darmstadt, Germany), and antimicrobial discs (Oxoid, Basingstoke, UK) were placed on the plate, incubated for 24 h at 37 °C. The inhibition zone was interpreted according to the Clinical Laboratory Standards Institute (CLSI, 2020) guidelines. The tested antibiotics were: penicillin (P–10U)—beta-lactam antibiotic, cefoxitin (FOX-30 μg)—beta-lactam antibiotic, gentamicin (CN-10 μg)—aminoglycosides, erythromycin (E-15 μg)—macrolides, tetracycline (TE-30 μg)—tetracyclines, ciprofloxacin (CIP-5 μg)—fluoroquinolones, nitrofurantoin (F-300 μg)—nitrofurantoins, clindamycin (DA-2 μg)—lincosamides, trimethoprim/sulfamethoxazole (SXT-1.25/23.75 μg)—sulfonamides, chloramphenicol (C-30 μg)—phenicols, rifampin (RD-5 μg)—ansamycins, quinupristin/dalfopristin (QD-15 μg)—streptogramins, linezolid (LZD-30 μg)—oxazolidinones and fusidic acid (FD-10 μg)—fusydates. *Staphylococcus aureus* ATCC 25923 reference strain was used for antimicrobial disc control.

### 2.3. Molecular Mechanisms of Antibiotic Resistance

Total genomic DNA of isolates was extracted using the Genomic Mini DNA isolation kit (A&A Biotechnology, Gdynia, Poland), following the manufacturer’s instructions. In all strains the presence of the genes: *mec*A, *tet*K and *tet*L genes encoding efflux pumps, actively removing antimicrobials from the cell; *tet*M (involved in the disruption of cell antibiotic transport and in modification of the drug target site) [15]; *mph(*C), *msr(*A/B), *erm*A, *erm*B, *erm*C genes (involved on resistance to macrolides and lincosamides); *vga*(A) (streptogramin A resistance gene) [9]; *bla*Z (encoding penicillinase) [16]; *mec*C [17], *fus*B, *fus*C, *fus*D genes (encoding low-level fusidic acid resistance) [18]; *aac(6′)-Ie-aph(2″)-Ia* (bifunctional aminoglycoside-modifying enzyme gene) [19]; the *aph(2″)-Ic* (aminoglycoside phosphotransferase (APH) enzyme gene) [20] were examined.

Both simple and multiplex PCR methods with specific primers and conditions were performed to gene presence (Table S1). Amplified DNAs were separated by electrophoresis in 1 × TBE buffer in a 1.5% agarose gels (Agarose Basica LE) stained by 0.5 μg/mL of ethidium bromide (0.5 mg/mL; Sigma-Aldrich) at 100 V/6 min followed by 80 V/90 min. Visualization was made using G-BOX F3 system (Syngene, UK) and Gene Tools program (Syngene, UK).

To explore the linezolid resistance, some resistance mechanisms were checked, such as: methylation of 23 SrRNA—*cfr* gene [21]; mutation in ribosomal proteins—*rpl* [22] and efflux—*opr*A [23], using primers listed in Table S1. The PCR products were treated with a Clean-Up purification kit (A&A Biotechnology, Gdańsk, Poland) and then sequenced by a commercial enterprise (Genomed, Warsaw, Poland). Five copies of the 23S rRNA gene were amplified using specific primers for each gene copy, as previously described by Pillai et al. [24]. Sequences of novel mutations were uploaded to GenBank.

### 2.4. Statistical Analysis

Antimicrobial resistance phenotypic profiles and gene occurrence were converted into numerical or binary coding for statistical analysis. Resistance to an antibiotic was represented as 1, and sensitivity was represented as 0. The same method was used for absence or presence of a specific gene; 0 and 1 were used, respectively. For standard statistical tests GraphPad Prism (GraphPad Software, San Diego, CA, USA) was used. At *p* < 0.05 results were considered statistically significant. The RStudio program was used for data visualization.

## 3. Results

### 3.1. Occurrence and Phenotypic Resistance in CoNS from Ready-to-Eat

Eighty-five CoNS strains, obtained from 198 RTE food samples, including salads, fresh squeeze juices, hamburgers, sushi, meat and fish tartars, obtained from 11 randomly selected restaurants and bars in Olsztyn, Poland, were investigated. Among the 85 CoNS isolates, 67 (78.8%) were resistant to at least one tested antibiotic, and among them 37 (43.5%) were multidrug resistant (resistant to three or more antibiotic classes). All multidrug resistance (MDR) combination patterns were summarized in Table S2b. In CoNS, the most common resistance was found against penicillin (48/85; 55.8%), followed by erythromycin (34/85; 40%), cefoxitin (31/85; 36.5%), clindamycin (29/85; 34.1%), fusidic acid (24/85; 28.2%), quinupristin/dalfopristin (21/85; 24.7%), gentamicin (19/85; 22.4%) and rifampicin (16/85; 18.8%). The resistance to the other tested antibiotics was found at lower frequency (4.7–1.2%). None of the isolates was found as resistant to ciprofloxacin (Table 1; Table S3). In Table 1 the antimicrobial-resistant phenotypes and genotypes of CoNS are shown, while more accurate results are summarized in Supplementary Tables S2a,b and S3.

**Figure 1:**
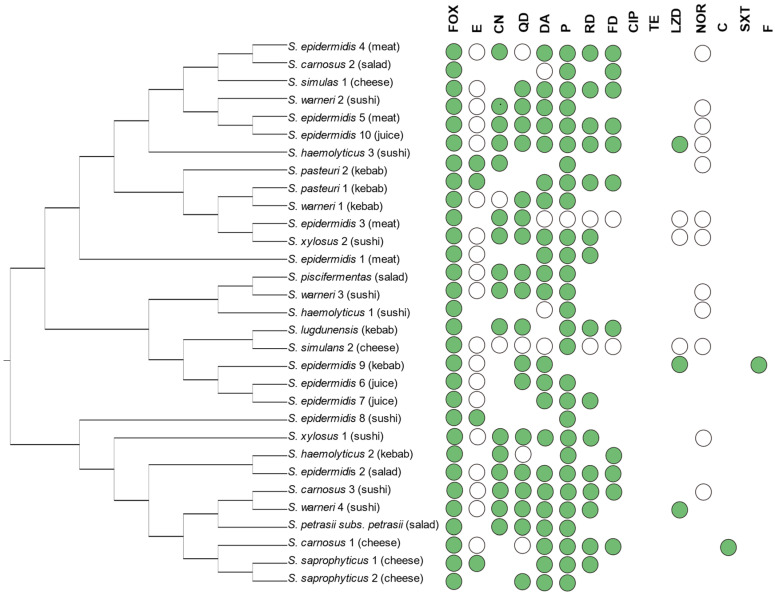
Antibiotic resistance profile of MR-CoNS isolates. Phylogenetic tree was obtained from the analysis of the partial gene sequence of *tuf* from isolates. Phylogenetic tree was created from DNA sequences using the neighbor-joining method with Kimura 2-parameter distance correction model with 1000 bootstrap replications using the MEGA X software. Abbreviations: green circles—resistant; circles without filling—medium sensitive, shape completely omitted-sensitive. P—penicillin, QD—quinupristin/dalfopristin, E—erythromycin, FOX—cefoxitin, DA—clindamycin, RD—rifampicin, FD—fusidic acid, CN—gentamycin, NOR—norfloxacin, F—nitrofurantoin, LZD—linezolid, TE—tetracycline, C—chloramphenicol, CIP—ciprofloxacin, SXT—trimethoprim/sulfamethoxazole. Strains antibiogram visualized using iTOL.

In details, 31 (36.5%) CoNS strains were found as phenotypically methicillin resistant (MR-CoNS), and all of them showed an MDR pattern (Table S2b). Nevertheless, all 31 strains resulted sensitive to sulfamethoxazole/trimethoprim, nitrofurantoin, tetracycline and ciprofloxacin (Figure 1).

Moreover, among the MR-CoNS isolates, the prevalence of resistance was as follows: cefoxitin (100%), penicillin (93.5%), quinupristin/dalfopristin (67.7%), gentamycin (58.1%), norfloxacin (32.3%), erythromycin (74.2%), clindamycin (74.2%), rifampicin (51.6%), fusidic acid (35.5%), linezolid (9.7%) and chloramphenicol (3.2%). Focusing on the belonging species, the MR-CoNS strains were mainly belonging to S. epidermidis (9/31, 29%) followed by *S. warneri* (4/31, 12.9%) and by *S. carnosus* and *S. heamolyticus* (3/31, 9.7%). As reported in Figure 1, the MR-CoNS strains were mainly isolated from meat, kebab, salads and sushi as well as fresh squished juices RTE samples (Figure 1).

### 3.2. Genotypic Antimicrobial Resistance in CoNS

Results of genotypic AR for CoNS tested strains are shown in Table 1. Overall, most of the strains were *bla*Z positive (72/85; 84.7%) followed, with a lesser extent, by *mec*A positive strains (29.4%), whereas no *mec*C positive strains were found.

Zooming on the resistance to aminoglycosides, results revealed that it was mediated mostly by the *aac*(6′)-*Ie-aph*(2″)-*Ia* (39/85; 45.9%) gene and much less frequently by *aph*(*2″*)-*Ic* (3/85; 3.5%), whereas no strains showed the *aph*(2″)-*Ic* gene. Macrolide resistance was mostly encoded by *msr*A/B (58/85; 68.2%), followed by *erm*B (34/85; 40%) and *mph*C (4/85; 4.7%) alone or in different combinations. Tetracycline resistance, which was detected only in three isolates, belonging to S. warneri, S. simulans and *S. heamolyticus* species, was revealed as mediated by *tet*K and/or *tet*M and/or *tet*L genes, with the *tet*K gene most frequently detected (27/85; 31.8%) followed by *tet*M (14/85; 16.5%) and *tet*L (2/85; 2.35%). The *fus*B/C/D gene was found as responsible of the acquired low-level fusidic acid resistance in 15 out of 85 (17.6%) isolates. The streptogramin resistance determinant *vga*A was found in 26 isolates (30.6%) of which 21 (24.7%) and showed phenotypic resistance to quinupristin/dalfopristin (QD) (Table 1). It is relevant to underline that, within the four strains phenotypically resistant to linezolid (LZD_R_), in three of them (2 *S. epidermidis* and 1 *S. warneri*) a mutation was detected, as revealed by L101V and V188I changes, in the L3 protein amino acid sequence [GenBank accession no. OQ028694; OQ028697; OQ058823]. The presence of the methyltransferase *cfr* gene and ABC-type transporter gene *opr*A gene were not identified in any of the LZD_R_ strain.

### 3.3. Correlation between Phenotype and Genotype Antimicrobial Resistance in CoNS from RTE Food

**Figure 2:**
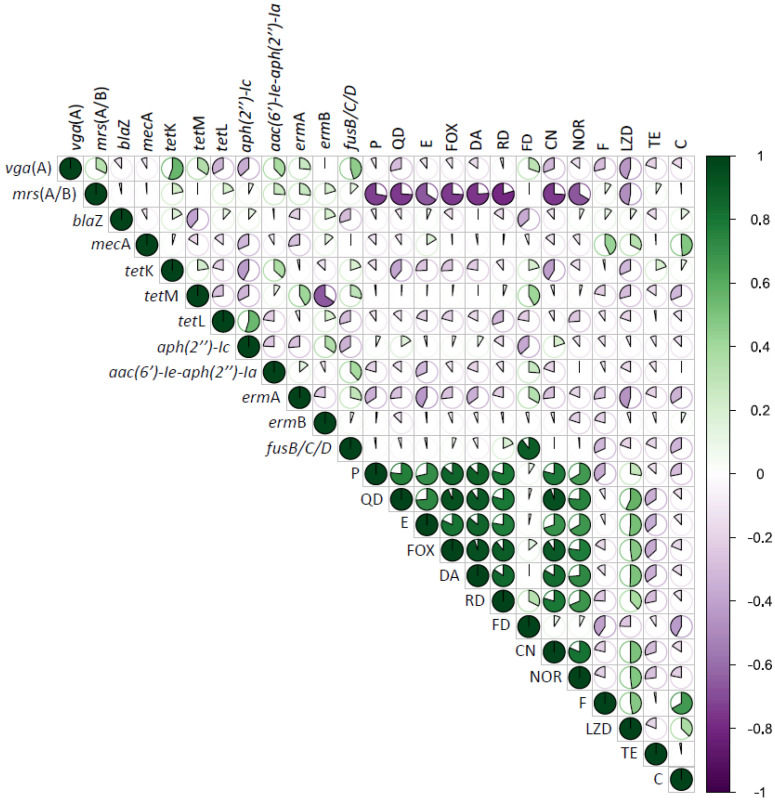
Correlation matrix of genotypic and phenotypic antibiotic resistance of all (*n* = 85) CoNS isolates. Green is positive correlation and purple is negative correlation. The strength of the color and size indicate the numerical value of correlation coefficient. Abbreviations: P—penicillin, QD—quinupristin/dalfopristin, E—erythromycin, FOX—cefoxitin, DA—clindamycin, RD—rifampicin, FD—fusidic acid, CN—gentamycin, NOR—norfloxacin, F—nitrofurantoin, LZD—linezolid, TE—tetracycline, C—chloramphenicol.

Correlation matrix analysis (Figure 2) was performed to determine associations between genotypic and phenotypic traits of CoNS strains. Significant positive correlations of AR revealed that the co-occurrence of resistance is prevalent and confirmed the presence of MDR isolates. For instance, resistance to the penicillin (β-lactam) was positively correlated (*p*  <  0.05) with resistance to quinupristin/dalfopristin, erythromycin, cefoxitin, clindamycin, rifampicin, fusidic acid, gentamycin, norfloxacin and linezolid (*p*  <  0.05). Alike, resistance to the macrolide erythromycin was positively correlated with resistance to antimicrobials combination: cefoxitin, clindamycin, rifampicin, fusidic acid, gentamycin, norfloxacin and linezolid (*p*  <  0.05). Notwithstanding, resistance to the tetracycline has not shown any positive correlations with any other antimicrobials.

Correlation analysis showed very few positive relationships between the presence of AR genes and the resistance to tested antibiotics (Figure 2, *p*  <  0.05). The presence of *mec*A gene was positively correlated with macrolide resistance (erythromycin and *erm*B gene), nitrofurantoin, linezolid and chloramphenicol. Any positive correlation between tetracyclines gene *tet*L presence and any other resistance was found.

## 4. Discussion

CoNSs are listed among the most common opportunistic microorganisms found in food. Nevertheless, Regulation 2073 Commission Regulation (EC) No 2073/2005 of 15 November 2005 on microbiological criteria for foodstuffs [25] does not refer any requirement to monitor CoNSs in food, as only the presence of *S. aureus* is subjected to control. Although such a legal status could be considered reasonable until a few years ago, when CoNS were regarded as less aggressive, now that their clinical significance has been recognized, it would be appropriate to adapt the regulations to the current state of knowledge. In this study, the presence of CoNS was evaluated in foods that are supposed to be directly consumed by consumers. In details, 85 non-duplicated CoNS strains isolated from RTE foods purchased in bars and restaurants in Poland were considered. In this type of food, the contaminations can originate not only from raw materials and/or ingredients but also from processing phases, during which both hygienic conditions and inappropriate human manipulations can have a considerable role. In the present study, as expected, the most common species was *S. epidermidis*, commonly found on the skin and mucous membranes of humans and animals and in their living environments. This result is in agreement with other findings which indicated *S. epidermidis* as the dominant species among staphylococci isolated from RTE meat products [26], cheeses [27] and fish [28]. Furthermore, these data are consistent with the increasing frequency of CoNs isolation from clinical specimens [29,30]. Actually, based on the results reported by Lee et al. [31], in 2018, *S. epidermidis* was declared a public health risk by the European Centre for Disease Prevention and Control.

The most alarming observation is the high occurrence of such a high percentage of AR strains, including MDR strains and, above all, MR-CoNS strains, isolated from RTE foods. Therefore, CoNS strains have been recognized as a direct hazard to public health, being vectors for AR gene spread to human potentially pathogenic bacteria, making vain the use of antibiotics for human health. It has been reported that MR-CoNS, found both in the food production environment and in final products, are capable of transferring the *mec*A gene to pathogenic *S. aureus* [32,33]. Genes encoding AR in staphylococci are usually located on plasmids, transposons or other mobile genetic elements (MGEs), offering a way for horizontal gene transfer [34]. Research focused on CoNS strains isolated from RTE foods has demonstrated that they present the same genes as those found in the genomes of pathogenic *S. aureus* isolated from production environment or clinical materials. Similar observations have been noted by other authors, e.g., in CoNS strains isolated from humans and animals, aquaculture [35], livestock farming [36], food industry and others, also in non-specific hosts [28]. Moreover, a study conducted in Greece on isolates from RTE food [37] demonstrated a high prevalence of MR-CoNS (41.7%). In Poland, 27.6% of CoNS isolated from poultry were found resistant to methicillin [38]. These AR-CoNS strains may be a major cause of infectious diseases due to their ability to form biofilms and, thus, to colonize community or hospital environments [32]. Recently, a study investigated the MR-SA colonization in HIV-infected patients revealing the role of MR-CoNS as a protective factor against MR-SA [39]. The study also observed a similarity in the profiles of resistance in MR-SA and MR-CoNS strains, which may reflect the MR-CoNS as a potential donor of antimicrobial resistance genes. In this context MR-CoNS with additional virulence factors was gaining more interest than MR-SA.

Furthermore, an alarming result of the present study is the high percentage of resistance to macrolides, lincosamides and streptogramin B (MLS_B_). Resistance to this group of antibiotics is determined by the expression of the *erm* and *msr* genes. Currently, MLS_B_ are preferred in the treatment of infections due to the increase in methicillin resistance, as an alternative for patients allergic to penicillin and due to their great pharmacokinetics. It is believed that staphylococcal strains simultaneously resistant to methicillin and MLS_B_ are among the most frequent causes of clinical infections [40]. Such infections are associated with increased mortality and, therefore, with prolonged hospitalization and increased treatment costs [41]. Within staphylococci, the resistance to macrolides has also been linked to cross-resistance to quaternary ammonium compounds (QAC) and cationic salts, with a broad range of activity against microorganisms, mainly against Gram-positive bacteria, widely used in ordinary environmental sanitation of noncritical surfaces in food production facilities. Previous studies revealed that the *qac* genes, encoding resistance to QACs, are located on the same MGEs (mostly plasmids), as the genes encoding resistance to macrolides [42]. It has also been concluded that the exposure of staphylococci to QACs, particularly at sub-lethal concentrations, induces resistance to macrolides. In particular, the QAC-induced overexpression of efflux pumps may induce cross-resistance to macrolides, increasing the risk of AR gene transferring [43].

In addition, the results of the present study pose a further concern, considering that the 44.2% of tested strains exhibited a multi-drug-resistance, defined as the lack of susceptibility to at least one agent in three or more chemical classes of antibiotic. The presence of MDR-CoNS in foods or food production environments poses the hazard of the spread of multiple resistance genes simultaneously to the human microbiota, including resistance to other pathogenic species. Consumption of food containing antimicrobial resistance genes may lead to their accumulation in the intestines or gastrointestinal tract [44]. As a result, people become reservoirs of resistance genes. The same applies to MDR bacteria, which can be transmitted to humans through food [45,46].

In the present study, among tested strains, LZD_R_ strains were also observed. LZD is an oxazolidinone antibiotic currently used to treat infections caused by Gram-positive cocci, especially MR-SA and vancomycin-resistant enterococci [47]. LZD is considered a drug of last resort, and its use is reserved for hospital practice only. The emergence of resistance to this antibiotic considerably narrows therapeutic options in cocci infections. According to previous reports, in the present study the dominant mechanism of resistance to LZD in CoNS strains was mutations in ribosomal protein L3 [48,49]. Interestingly, LZD resistance has emerged in *S. epidermidis, S. warneri* and *S. xylosus*. Among CoNS, the species *S. xylosus*, similar to *S. carnosus, S. condimenti, S. equorum, S. succinus* and *S. piscifermentans*, plays an important role in food production. Strains of *S*. *xylosus* and *S. carnosus* are used as starter cultures in production of fermented meat products (such as meat or salami sausages). It appears, however, that staphylococci belonging to these species also exhibit resistance to antibiotics, including those of major clinical importance.

## 5. Conclusions

The present study confirmed that food might be an important vector for the transmission of AR staphylococci. Although CoNS are not considered as food contaminants, results of the present study confirmed their occurrence in RTE food. In addition, the high frequency of AR among tested CoNS highlighted that this group of staphylococci may represent a reservoir of AR genes to different antibiotic classes, most commonly to β-lactams, tetracyclines, penicillins and macrolides. The high frequency of MR-CoNS constitutes a direct risk to public health as they increase the gene pool from which pathogenic bacteria can pick up resistance traits. Moreover, results on AR of *S. xylosus* and *S. carnosus* and against many different antibiotic groups impose specific selection criteria for strains to be used as starter cultures in the production of fermented meat products. CoNS resistance to antimicrobials used routinely requires strengthening forces in the development of resistance management strategies to continue the effective and safe use of antibiotics, especially in the treatment of critical diseases.

## Figures and Tables

**Table 1:** Antimicrobial resistance profiles of CoNS isolated from RTE food.

Species	No. of Isolates (%)	Antimicrobial Resistance	
		Phenotype	Genotype
*S. epidermidis*	21 (24.7%)	CN^(4)^, DA^(9)^, E^(16)^, FOX^(10)^, FD^(6)^, LZD^(2)^, NOR^(3)^, P^(13)^, RD^(6)^, QD^(5)^	*aac(6′)-Ie-aph(2″)-Ia^(11)^, bla*Z^(17)^, *erm*A^(8)^, *erm*B^(11)^, *fus*B/C/D^(5)^, *mec*A^(11)^, *mph(*C)^(3)^, *msr*(A/B)^(15)^, *tet*K^(7)^, *tet*L^(2)^, *tet*M^(3)^, *vga*(A)^(7)^
*S. warneri*	14 (16.5%)	C^(1)^, CN^(4)^, DA^(5)^, E^(4)^, FOX^(4)^, FD^(2)^, LZD^(1)^, NOR^(1)^, P^(8)^,TE^(1)^, RD^(2)^, QD^(4)^	*aac(6′)-Ie-aph(2″)-Ia^(7)^, bla*Z^(13)^, *erm*B^(3)^, *fus*B/C/D^(2)^, *mec*A^(1)^, *msr*(A/B)^(6)^, *tet*K^(6)^, *tet*M^(1)^, *vga*(A)^(4)^
*S. carnosus*	9 (10.6%)	CN^(1)^, DA^(3)^, E^(3)^, FOX^(3)^, FD^(4)^, NOR^(1)^, P^(3)^, RD^(2)^, QD^(3)^	*aac(6′)-Ie-aph(2″)-Ia^(5)^, aph(2″)-Ic^(1)^, bla*Z^(9)^, *erm*A^(3)^, *erm*B^(6)^, *fus*B/C/D^(2)^, *mec*A^(3)^, *mph(*C)^(1)^, *msr*(A/B)^(8)^, *tet*K^(5)^, *tet*M^(2)^, *vga*(A)^(3)^
*S. simulans*	9 (10.6%)	CN^(1)^, DA^(1)^, E^(1)^, FOX^(2)^, FD^(3)^, P^(4)^, RD^(2)^, TE^(1)^, QD^(1)^	*aac(6′)-Ie-aph(2″)-Ia^(3)^, bla*Z^(6)^, *erm*A^(5)^, *erm*B^(1)^, *fus*B/C/D^(3)^, *msr*(A/B)^(6)^, *tet*K^(1)^, *tet*M^(2)^, *vga*(A)^(5)^
*S. xylosus*	8 (9.4%)	CN^(2)^, DA^(3)^, E^(2)^, FOX^(2)^, FD^(1)^, LZD^(1)^, NOR^(2)^, P^(3)^, RD^(1)^, QD^(2)^	*aac(6′)-Ie-aph(2″)-Ia^(5)^, bla*Z^(8)^, *erm*A^(4)^, *erm*B^(3)^, *fus*B/C/D^(1)^, *mec*A^(1)^, *msr*(A/B)^(5)^, *vga*(A)^(2)^
*S. saprophyticus*	6 (7.1%)	DA^(3)^, E^(3)^, FOX^(2)^, FD^(2)^, P^(4)^, RD^(1)^, QD^(1)^	*aac(6′)-Ie-aph(2″)-Ia^(2)^, bla*Z^(4)^, *erm*A^(3)^, *mec*A^(1)^*, msr*(A/B)^(5)^, *tet*K^(3)^, *tet*M^(5)^, *vga*(A)^(2)^
*S. pasteuri*	5 (5.9%)	CN^(1)^, DA^(2)^, E^(3)^, FOX^(2)^, FD^(2)^, P^(5),^, RD^(1)^, QD^(1)^	*bla*Z^(5)^, *erm*A^(2)^, *erm*B^(2)^, *mec*A^(2)^, *msr*(A/B)^(2)^, *tet*K^(2)^, *tet*M^(1)^
*S. heamolyticus*	4 (4.7%)	CN^(2)^, DA^(1)^, E^(1)^, FOX^(3)^, FD^(2)^, NOR^(2)^, P^(4)^, TE^(1)^, QD^(1)^	*aac(6′)-Ie-aph(2″)-Ia^(2)^, bla*Z^(3)^, *erm*A^(2)^, *erm*B^(2)^, *mec*A^(3)^, *msr*(A/B)^(4)^, *tet*K^(1)^,
*S. petrasii* subsp. *petrasii*	4 (4.7%)	C^(2)^, CN^(1)^, DA^(1)^, F^(1)^, FOX^(1)^, P^(1)^, QD^(1)^	*aac(6′)-Ie-aph(2″)-Ia^(2)^, aph(2″)-Ic^(1)^, bla*Z^(4)^, *erm*B^(3)^, *mec*A^(2)^, *msr*(A/B)^(4)^, *tet*K^(2)^
*S. lentus*	2 (2.4%)	P^(1)^	*aac(6′)-Ie-aph(2″)-Ia^(1)^, bla*Z^(2)^, *erm*A^(1)^, *erm*B^(1)^, *mec*A^(1)^, *msr*(A/B)^(1)^
*S. piscifermentas*	2 (2.4%)	CN^(1)^, DA^(1)^, E^(1)^, FOX^(1)^, FD^(1)^, P^(1)^, QD^(1)^	*aph(2″)-Ic^(1)^, bla*Z^(1)^, *erm*B^(2)^, *fus*B/C/D^(1)^, *msr*(A/B)^(2)^
*S. lugdenensis*	1 (1.2%)	CN^(1)^, FOX^(1)^, FD^(1)^, P^(1)^, QD^(1)^, RD^(1)^,	*erm*A^(1)^
Total	85	C^(3)^, CN^(19)^, DA^(29)^, E^(34)^, F^(1)^, FD^(24)^, FOX^(31)^, LZD^(4)^, NOR^(9)^, P^(48)^, QD^(21)^, RD^(16)^, TE^(3)^	*aac(6′)-Ie-aph(2″)-Ia^(39)^, aph(2″)-Ic^(3)^, bla*Z^(72)^, *erm*A^(29)^, *erm*B^(34)^, *fus*B/C/D^(15)^, *mec*A^(25)^, *mph(*C)^(4)^, *msr*(A/B)^(58)^, *tet*K^(27)^, *tet*L^(2)^, *tet*M^(14)^, *vga*(A)^(26)^

Abbreviations: C—chloramphenicol, CN—gentamicin, DA—clindamycin, E—erythromycin, F—nitrofurantoin, FD—fusidic acid, FOX—cefoxitin, LZD—linezolid, NOR—norfloxacin, P—penicillin, QD—quinupristin/dalfopristin, RD—rifampicin, TE—tetracycline. Note: the superscript number in parentheses after gene and antibiotic indicates, respectively, the number of strains carrying that gene and the number of strains resistant to that antibiotic.

## Data Availability

Data is contained within the article or supplementary material.

## Supplementary Materials

The following supporting information can be downloaded at: https://www.mdpi.com/article/10.3390/foods12030514/s1, Table S1: Primers used for screening of resistance determinants in CoNS.; Table S2a: Antibiotic resistance profiles in *Staphylococcus* spp. isolated from ready-to-eat food; Table S2b: Antibiotic resistance profiles of MDR-CoNS isolated from ready-to-eat food; Table S3: Percentage of *Staphylococcus* spp. strains resistant to different antibiotics; Table S4: Results of antibiotic resistance genes presence depending on the species (References [9,16,17,18,19,20,21,22,23,50,51,52] are cited in Supplementary Materials).
