# Supplementary material for: A Comprehensive Study on Antibiotic Resistance among Coagulase-Negative Staphylococci (CoNS) Strains Isolated from Ready-to-Eat Food Served in Bars and Restaurants
Source: Foods. 2023 Jan 23;12(3):514. doi: 10.3390/foods12030514 (PMC9914766; doi:10.3390/foods12030514)
Supplement: Supplementary file 1 [file foods-12-00514-s001.zip › foods-2156651-supplementary.pdf]

foods

Table S1. Primers used for screening of resistance determinants in *CoNS*.

Antimicrobial	Gene	Primer sequence 5′-3′	Amplicon size (bp)	References
Penicillin	*blaZ*	F: CAAAGATGATATAGTTGCTTATTCTC<br>R: TGCTTGACCACTTTTATCAGC	400	[1]
Oxacillin	*mec*A	F: AAAATCGATGGTAAAGGTTGGC<br>R: AGTTCTGGCACTACCGGATTTGC	533	[2]
	mecC	F F: GAAAAAAAGGCTTAGAACGCCTC<br>R: GAAGATCTTTTCCGTTTTCAGC	138	[3]
Tetracycline	*tet*K	F: TTATGGTGGTTGTAGCTAGAAA<br>R: AAAGGGTTAGAAACTCTTGAAA	348	[4]
	*tet*M	F: GTGGACAAAGGTACAACGAG<br>R: CGGTAAAGTTCGTCACACAC	406	[5]
	*tet*L	F: TGGTGGAATGATAGCCCATT<br>R: CAGGAATGACAGCACGCTAA	229	[4]
Fusidic acid	*fus*B	F: CCGTCAAAGTTATTCAATCG<br>R: ACAATGAATGCTATCTCGACA	492	[6]
	*fus*C	F: GGACTTTATTACATCGATTGAC<br>R: CTGTCATAACAAATGTAATCTCC	411	
	fusD	F: AATTCGGTCAACGATCCC<br>R: GCCATCATTGCCAGTACG	465	
Macrolides	*msr*(A/B)	F: TCCAATCATAGCACAAAATC<br>R: AATTCCCTCTATTTGGTGGT	162	[7]
	mph(C)	F: ATGACTCGACATAATGAAAT<br>R: CTACTCTTTCATACCTAACTC	900	
	*erm*A	F: TCTAAAAAGCATGTAAAAGAA<br>R: CTTCGATAGTTTATTAATATTAG	645	
	*erm*B	F: GAAAAGTACTCAACCAAATA<br>R: AGTAACGGTACTTAAATTGTTTA	639	
	*erm*C	F: TCAAAACATAATATAGATAAA<br>R: GCTAATATTGTTTAAATCGTCAAT	642	
Streptogramins	*vga*(A)	F: AGTGGTGGTGAAGTAACACG<br>R: GGTTCAATACTCAATCGACTGAG	1264	
Aminoglycosides	*aac(6′)-Ie-aph(2′′)-Ia*	F: CAGGAATTTATCGAAAATGGTAGAAAAG<br>R: CACAATCGACTAAAGAGTACCAATC	369	[8]
	aph(2′′)-Ic	F: ATACAATCCGTCGAGTCGCT<br>R: GTTGGCCTTATCCTCTTCCA	837	[9]
Oxazolidinone	*cfr*	F: TGAAGTATAAAGCAGGTTGGGAGTCA<br>R: ACCATATAATTGACCACAAGCAGC	746	[10]
	*rpl*C	F: GCGCTTCATTCGTGAATTCAA<br>R: TTCTTTCTGCATCGACACGTACAA		[11]
	*rpl*D	F: ACGATGCAATCGTAATGCAA<br>R: TTCAGCAACTTTTTCTGACAA		
	*rpl*V	F: GGACATGCTGCTGACGATA<br>R: ACCATTTAGCATCCCAGTCG		
	*opr*A	F: F: TACTTGATGAACCTACTAACCA<br>R: CCTTGAACTACTGATTCTCGG	422	[12]

Table S2a. Antibiotic resistance profiles in *Staphylococcus* spp. isolated from ready-to-eat food.

Species	Phenotyping resistance profiles	Number (%) of strains	Number of antibiotics
S. carnosus (n=9)	P, CN, QD, NOR*, E*, FOX, DA, RD, FD	1 (11.1)	9
	P, QD*, E*, FOX, DA, RD, FD	1 (11.1)	7
	P, FOX, DA*, FD	1 (11.1)	4
	E, FD	1 (11.1)	2
	QD*	1 (11.1)	1
	-	4 (44.4)	0
S. epidermidis (n=21)	LZD, P, CN, QD, NOR*, E*, FOX, DA, RD, FD	1(4.8)	10
	P, CN, QD*, NOR*, E*, FOX, DA, RD, FD	1(4.8)	9
	P, CN, QD, NOR*, E*, FOX, DA, RD, FD	1(4.8)	8
	P, CN, QD, E*, FOX, DA, RD	2 (9.5)	7
	F, LZD, QD, E*, FOX, DA	1(4.8)	6
	P, E*, FOX, DA, RD	1(4.8)	5
	P, QD, E*, FOX, DA	1(4.8)	5
	P, E, DA	1(4.8)	3
	P, E, FOX	1(4.8)	3
	E*, FOX, FD	1(4.8)	3
	P, E*	1(4.8)	2
	P, FD	1(4.8)	2
	E, FD	3 (14.3)	1
	E*	2 (9.5)	1
	-	3 (14.3)	0
S. haemolyticus (n=4)	P, CN, NOR*, E, FOX	1 (25)	5
	P, CN, QD*, FOX, FD	1 (25)	5
	P, NOR*, FOX, DA*	1 (25)	4
	P, TE, FD	1 (25)	3
S. lentus (n=2)	P	1 (50)	1
	-	1 (50)	0
S. lugdenensis (n=1)	P, CN, QD, FOX, RD, FD	1 (100)	6
S. pasteuri (n=5)	P, E, FOX, DA, RD, FD	1 (20)	6
	P, CN*, QD, E*, FOX, DA	1 (20)	6
	FD	2 (20)	1
	P	1 (20)	1
	E	1 (20)	1
S. petrasii subsp. petrasii (n=4)	P, CN, QD, FOX, DA	1 (25)	5
	F, C*	1 (25)	2
	C	1 (25)	1
	-	1 (25)	0
S. piscifermrntans (n=2)	P, CN, QD, E*, FOX, DA	1 (50)	6
	FD	1 (50)	1
S. saprophyticus (n=6)	P, E, FOX, DA, RD, FD	1 (16.7)	6
	P, QD, FOX, DA	1 (16.7)	4
	P, E*, DA	1 (16.7)	3
	E, FD	1 (16.7)	2
	P	1 (16.7)	1
	-	1 (33.3)	0
S. simulans (n=9)	P, QD, E*, FOX, DA, RD	1 (11.1)	6
	P, CN*, FOX, RD*, FD	1 (11.1)	5
	P, FD	1 (11.1)	2
	TE*	1 (11.1)	1
	P	2 (22.2)	1
	-	4 (44.4)	0

S. warneri (n=14)	LZD, P, C, CN, QD, E, FOX, DA, RD	1 (7.1)	9
	P, CN, QD*, E*, FOX, DA, RD	1 (7.1)	7
	P, CN, QD, NOR*, E*, FOX, DA	1 (7.1)	7
	P, CN, QD, FOX, DA	1 (7.1)	5
	P, E, DA*	1 (7.1)	3
	P, TE	1 (7.1)	2
	P	2 (14.9)	1
	FD	2 (14.9)	1
	-	4 (28.6)	0
S. xylosus (n=8)	P, CN, QD, NOR*, E*, FOX, DA, RD	1 (12.5)	8
	LZD, P, CN, QD, NOR*, E*, FOX, DA	1 (12.5)	8
	P, DA*, FD	1 (12.5)	3
	P	1 (12.5)	1
	-	4 (50)	0

Abbrevations: *intermediate resistance; n= number of MR-CoNS isolated; penicillin (P–10U) - penicillinase, cefoxitin (FOX-30 μg) - penicillinase, gentamicin (CN-10 μg) - aminoglycosides, erythromycin (E-15 μg) - macrolides, tetracycline (TE30 μg) - tetracyclines, ciprofloxacin (CIP-5 μg) - fluoroquinolones, nitrofurantoin (F-300 μg) - nitrofurantoins, clindamycin (DA-2 μg) - lincosamides, trimethoprim/sulfamethoxazole (SXT-1.25/23.75 μg) – folate pathway inhibitors, chloramphenicol (C-30 μg) - phenicols, rifampin (RD-5 μg) - ansamycins, quinupristin/dalfopristin (QD-15 μg) – streptogramins and linezolid (LZD-30 μg) – oxazolidin

Table S2b. Antibiotic resistance profiles of multidrug-resistant CoNS isolated from ready-to-eat food.

Species	Phenotyping resistance profiles	Number (%) of strains	Number of antibiotics
S. carnosus (n=3)	P, CN, QD, NOR*, E*, FOX, DA, RD, FD	1 (33.3)	9
	P, QD*, E*, FOX, DA, RD, FD	1 (33.3)	7
	P, FOX, DA*, FD	1 (33.3)	4
S.epidermidis (n=9)	LZD, P, CN, QD, NOR*, E*, FOX, DA, RD, FD	1 (11.1)	10
	P, CN, QD*, NOR*, E*, FOX, DA, RD, FD	1 (11.1)	9
	P, CN, QD, NOR*, E*, FOX, DA, RD, FD	1 (11.1)	9
	P, CN, QD, E*, FOX, DA, RD, FD	1 (11.1)	8
	F, LZD, QD, E*, FOX, DA	1 (11.1)	6
	P, E*, FOX, DA, RD	1 (11.1)	5
	P, QD, E*, FOX, DA	1 (11.1)	5
	P, E*, FOX, DA, RD	1 (11.1)	5
	E*, FOX, FD	1 (11.1)	3
	P, E, FOX	1 (11.1)	3
S. heamolyticus (n=3)	P, CN, QD*, FOX, FD	1 (33.3)	5
	P, CN, NOR*, E, FOX	1 (33.3)	5
	P, NOR*, FOX, DA*	1 (33.3)	4
S. lugdenensis (n=1)	P, CN, QD, FOX, RD, FD	1 (100)	6
S. pasteuri (n=2)	P, CN*, QD, E*, FOX, DA	1(50)	6
	P, E, FOX, DA, RD, FD	1(50)	5
S. petrasii subsp. petrasii (n=1)	P, CN, QD, FOX, DA	1 (100)	5
S. piscifermrntans (n=1)	P, CN, QD, E*, FOX, DA	1 (100)	6
S. saprophyticus (n=2)	P, E, FOX, DA, RD	1(50)	5
	P, QD, FOX, DA	1(50)	4
S. simulans (n=2)	P, QD, E*, FOX, DA, RD,FD	1(50)	7
	P, CN*, FOX, RD*, FD	1(50)	5
S. warneri (n=4)	P, CN, QD, FOX, DA	1(25)	5
	P, CN, QD, NOR*, E*, FOX, DA	1(25)	7
	P, CN, QD*, E*, FOX, DA, RD	1(25)	7
	LZD, P, C, CN, QD, E, FOX, DA, RD	1(25)	9
S. xylosus (n=2)	P, CN, QD, NOR*, E*, FOX, DA, RD	1(50)	8
	LZD, P, CN, QD, NOR*, E*, FOX, DA	1(50)	8

Abbrevations: *intermediate resistance; n= number of MR-CoNS isolated; penicillin (P–10U) - penicillinase, cefoxitin (FOX-30 μg) - penicillinase, gentamicin (CN-10 μg) - aminoglycosides, erythromycin (E-15 μg) - macrolides, tetracycline (TE30 μg) - tetracyclines, ciprofloxacin (CIP-5 μg) - fluoroquinolones, nitrofurantoin (F-300 μg) - nitrofurantoins, clindamycin (DA-2 μg) - lincosamides, trimethoprim/sulfamethoxazole (SXT-1.25/23.75 μg) – folate pathway inhibitors, chloramphenicol (C-30 μg) - phenicols, rifampin (RD-5 μg) - ansamycins, quinupristin/dalfopristin (QD-15 μg) – streptogramins and linezolid (LZD-30 μg) – oxazolidino

Table S3. Percentage of *Staphylococcus* spp. strains resistant to different antibiotics.

Antimicrobial agent			*Staphylococcus sp.* (n=85)			
			R (n)	R (%)	S (n)	S (%)
Penicillin	P	10	48	55.8	37	43.5
Erythromy-	E	15	34	40. 0	51	60. 0
Cefoxitin	FOX	30	31	36.5	54	62.8
Clindamy-	DA	2	29	34.1	56	65.9
Fusidic acid	FD	10	24	28.2	61	71.8
Quinupris-	QD	15	21	24.7	64	75.3
Gentamicin	CN	120	19	22.4	66	77,6
Rifampicin	RD	5	16	18.8	69	81.1
Linezolid	LZD	30	3	3.5	82	96.5
Chloram-	C	30	3	3.5	82	96.5
Tetracycline	T	30	3	3.5	82	96.5
Nitrofu-	F	300	1	1.2	84	98.8
Ciprofloxa-	CIP	5	0	0	85	100
Trime-thoprim/sul-famethoxa-zole	SXT	1.25/23.75	0	0	85	100

Table S4. Results of antibiotic resistance genes presence depending on the species.

Species	No. of isolates	Number (%) of strains											
		blaZ	*mecA*	*nuc*	*tetK*	*tetL*	*tetM*	*ermB*	*msrA/B*	*aac (6′)-Ie aph (2″)-Ia*	*fusB/C/D*	*vgaA*	*mphC*
S. epidermidis	21	17 (81.0)	11 (52.4)	13 (61.9)	7 (33.3)	2 (9.5)	3 (14.9)	11 (52.4)	15 (71.4)	11 (52.4)	5 (23.8)	7 (33.3)	3 (14.9)
S. warneri	14	13 (92.9)	1 (7.1)	9 (64.3)	6 (42.9)	0	1 (7.1)	3 (21.4)	6 (42.9)	7 (50)	2 (14.3)	4 (28.6)	0
S. carnosus	9	9 (100)	3 (22.2)	9 (100)	5 (55.5)	0	2 (22.2)	6 (66.7)	8 (88.9)	5 (55.5)	2 (22.2)	3 (33.3)	1 (11.1)
S. simulans	9	6 (66.7)	0	0	1 (11.1)	0	2 (22.2)	1 (11.1)	6 (66.7)	3 (33.3)	3 (33.3)	5 (55.6)	0
S. xylosus	8	8 (100)	1 (12.5)	6 (75.0)	0	0	0	3 (37.5)	5 (62.5)	5 (62.5)	1 (12.5)	2 (25)	0
S. saprophyticus	6	4 (66.7)	1 (16.7)	5 (83.3)	3 (50)	0	5 (83.3)	0	5 (83.3)	2 (33.3)	0	2 (33.3)	0
S. pasteuri	5	5 (100)	2 (40.0)	3 (60.0)	2 (40)	0	1 (20)	2 (40)	2 (40)	0	0	0	0
S. heamolyticus	4	3 (75.0)	3 (75.0)	1 (25.0)	1 (25)	0	0	2 (50)	4 (100)	2 (50)	1 (25)	0	0
S. petrasii subsp. petrasii	4	4 (100)	2 (50.0)	4 (100)	2 (50)	0	0	3 (75)	4 (100)	2 (50)	0	0	0
S. lentus	2	2 (100)	1 (50.0)	0	0	0	0	1 (50)	1(50)	1 (50)	0	0	0
S. piscifermentas	2	1 (50.0)	0	1 (50.0)	0	0	0	2 (100)	2 (100)	0	1 (50.0)	0	0
S. lugdenensis	1	0	0	1 (100)	0	0	0	0	0	0	0	0	0
**Total**	**85**	**72 (84,7)**	**25 (29,4)**	**53 (62.4)**	**27 (31.8)**	**2 (2.35)**	**14 (16.5)**	**34 (40.0)**	**58(68.2)**	**39 (45.9)**	**15(17.6)**	**26 (30.6)**	**4 (4.7)**

1. Kaase, M., Lenga, S., Friedrich, S., Szabados, F., Sakinc, T., Kleine, B., & Gatermann, S. G. (2008). Comparison of phenotypic methods for penicillinase detection in Staphylococcus aureus. *Clinical Microbiology and Infection*, *14*(6), 614–616. https://doi.org/10.1111/j.1469-0691.2008.01997.x
2. Barski, P., Piechowicz, L., Galiński, J., & Kur, J. (1996). Rapid assay for detection of methicillin-resistant staphylococcus aureus using multiplex PCR. *Molecular and Cellular Probes*, *10*(6), 471–475. https://doi.org/10.1006/mcpr.1996.0066
3. Nelli, A.; Voidarou, C.; Venardou, B.; Fotou, K.; Tsinas, A.; Bonos, E.; Fthenakis, G.C.; Skoufos, I.; Tzora, A. Antimicrobial and Methicillin Resistance Pattern of Potential Mastitis-Inducing *Staphylococcus aureus* and Coagulase-Negative Staphylococci Isolates from the Mammary Secretion of Dairy Goats. *Biology* **2022**, *11*, 1591. https://doi.org/10.3390/biology11111591
4. Gevers, D., Danielsen, M., Huys, G., & Swings, J. (2003). Molecular characterization of tet(M) genes in Lactobacillus isolates from different types of fermented dry sausage. *Applied and Environmental Microbiology*, *69*(2), 1270–1275. https://doi.org/10.1128/AEM.69.2.1270-1275.2003
5. Roberts, M. C. (1994). Epidemiology of tetracycline-resistance determinants. *Trends in Microbiology*, *2*(10), 353–357. https://doi.org/10.1016/0966-842X(94)90610-6
6. Hritcu, O. M., Schmidt, V. M., Salem, S. E., Maciuca, I. E., Moraru, R. F., Lipovan, I., Mareş, M., Solcan, G., & Timofte, D. (2020). Geographical Variations in Virulence Factors and Antimicrobial Resistance Amongst Staphylococci Isolated From Dogs From the United Kingdom and Romania. *Frontiers in Veterinary Science*, *7*(July), 1–10. https://doi.org/10.3389/fvets.2020.00414
7. Silva, V., Caniça, M., Ferreira, E., Vieira-Pinto, M., Saraiva, C., Pereira, J. E., Capelo, J. L., Igrejas, G., & Poeta, P. (2022). *Multidrug-Resistant Methicillin-Resistant Coagulase-Negative Staphylococci in Healthy Poultry Slaughtered for Human Consumption*. *11*, 365. https://doi.org/10.3390/antibiotics11030365
8. Padmasini, E., Padmaraj, R., & Ramesh, S. S. (2014). High level aminoglycoside resistance and distribution of aminoglycoside resistant genes among clinical isolates of Enterococcus species in Chennai, India. *The Scientific World Journal*, *2014*. https://doi.org/10.1155/2014/329157
9. Kobayashi, N., Mahbub Alam, M., Nishimoto, Y., Urasawa, S., Uehara, N., & Watanabe, N. (2001). Distribution of aminoglycoside resistance genes in recent clinical isolates of Enterococcus faecalis, Enterococcus faecium and Enterococcus avium. *Epidemiology and Infection*, *126*(2), 197–204. https://doi.org/10.1017/S0950268801005271
10. Kehrenberg C., Cuny C., Strommenger B., Schwarz S., Witte W. (2009). Methicillin-resistant and -susceptible Staphylococcus aureus strains of clonal lineages ST398 and ST9 from swine carry the multidrug resistance gene cfr. Antimicrob. Agents Chemother. 53 779–781. 10.1128/aac.01376-08-1378
11. Mališová, L.; Jakubů, V.; Pomorská, K.; Musílek, M.; Žemličková, H. Spread of Linezolid-Resistant Enterococcus spp. in Human Clinical Isolates in the Czech Republic. Antibiotics 2021, 10, 219
12. Brenciani, A.; Morroni, G.; Vincenzi, C.; Manso, E.; Mingoia, M.; Giovanetti, E.; Varaldo, P.E. Detection in Italy of two clinical Enterococcus faecium isolates carrying both the oxazolidinone and phenicol resistance gene optrA and a silent multiresistance gene cfr. J. Antimicrob. Chemother. 2016, 71, 1118–1119.
